# Targeting *ABCB1 (MDR1)* in multi-drug resistant osteosarcoma cells using the CRISPR-Cas9 system to reverse drug resistance

**DOI:** 10.18632/oncotarget.13148

**Published:** 2016-11-07

**Authors:** Tang Liu, Zhihong Li, Qing Zhang, Karen De Amorim Bernstein, Santiago Lozano-Calderon, Edwin Choy, Francis J. Hornicek, Zhenfeng Duan

**Affiliations:** ^1^ Department of Orthopaedics, The 2^nd^ Xiangya Hospital of Central South University, Changsha, Hunan, 410011, P.R. China; ^2^ Sarcoma Biology Laboratory, Department of Orthopaedic surgery, Massachusetts General Hospital and Harvard Medical School, Boston, Massachusetts 02114, USA

**Keywords:** osteosarcoma, CRISPR-Cas9, meta-analysis, ABCB1, P-glycoprotein

## Abstract

**Background:**

Multi-drug resistance (MDR) remains a significant obstacle to successful chemotherapy treatment for osteosarcoma patients. One of the central causes of MDR is the overexpression of the membrane bound drug transporter protein P-glycoprotein (P-gp), which is the protein product of the MDR gene *ABCB1*. Though several methods have been reported to reverse MDR *in vitro* and *in vivo* when combined with anticancer drugs, they have yet to be proven useful in the clinical setting.

**Results:**

The meta-analysis demonstrated that a high level of P-gp may predict poor survival in patients with osteosarcoma. The expression of P-gp can be efficiently blocked by the clustered regularly interspaced short palindromic repeats (CRISPR)-associated Cas9 system (CRISPR-Cas9). Inhibition of *ABCB1* was associated with reversing drug resistance in osteosarcoma MDR cell lines (KHOSR2 and U-2OSR2) to doxorubicin.

**Materials and Methods:**

We performed a meta-analysis to investigate the relationship between P-gp expression and survival in patients with osteosarcoma. Then we adopted the CRISPR-Cas9, a robust and highly efficient novel genome editing tool, to determine its effect on reversing drug resistance by targeting endogenous *ABCB1* gene at the DNA level in osteosarcoma MDR cell lines.

**Conclusion:**

These results suggest that the CRISPR-Cas9 system is a useful tool for the modification of *ABCB1* gene, and may be useful in extending the long-term efficacy of chemotherapy by overcoming P-gp-mediated MDR in the clinical setting.

## INTRODUCTION

Osteosarcoma is one of the most common malignant tumors of bone, which mainly affects children and adolescents [1]. Current treatment for osteosarcoma involves surgical resection and multi-agent chemotherapy [1]. The advancement in intensive chemotherapy has significantly improved the 5-year survival rate from 20% with surgery alone to approximately 60-70% when combined with chemotherapy [2]. However, systemic relapses still occur in 40% of patients, which are mostly linked to chemotherapy drug resistance [3]. Increasing drug dosage in histologically poor responders has not improved their outcome [4]. Nearly 50% of osteosarcoma cases are either resistant to chemotherapy or acquire resistance during treatment [5].

Overcoming drug resistance is one approach to improving the survival rate of osteosarcoma patients [6]. The development of drug resistance is associated with multiple mechanisms. One of the major causes of multi-drug resistance (MDR) is the overexpression of the membrane bound drug transporter protein P-glycoprotein (P-gp) [7, 8]. P-gp is the protein product of the MDR gene *ABCB1* (*ABC* subfamily B member 1, also know as *MDR-1*) and acts as an energy-dependent drug efflux pump that requires two ATPs to pump out many structurally unrelated chemotherapeutic drugs [8]. A direct correlation between P-gp expression levels and the degree of drug resistance has been established in osteosarcoma cell lines [7, 9]. Several studies have tried to investigate the relevance of P-gp expression in osteosarcoma progression, but the results remain controversial [10–21]. For example, a previous study showed there was no correlation between MDR1 mRNA expression and disease progression in patients with osteosarcom [10]. However, another study found positive immuno-staining for P-gp is an independent risk factor for a poor outcome of osteosracoma patients [21]. In our previous study, we also found expression of P-gp is significantly predictor in patients with stage IIB osteosarcoma [17]. These data suggest that further study is required to clarify the prognostic value of P-gp expression in osteosarcoma. Meta-analysis uses a statistical approach that systematically combines the results from previous multiple research studies to obtain a conclusion [22].

Strategies for reversing and preventing MDR by targeting *ABCB1* have been studied extensively in different MDR model systems, including in osteosarcoma, but have shown limited clinical potential [7–9]. A possible approach to circumvent MDR is the co-administration of inhibitors/compounds that inhibit the transport activity of MDR transporters [7, 9, 23]. Four generations of P-gp inhibitors have been developed [24], including verapamil, cyclosporine A (CsA), reserpine, dexverapamil, PSC-883, VX710, XR9576 (tariquidar), R101933 (laniquidar), flavonoids, and a natural product, curcumin [25]. Other substances, such as a new synthetic rifampicin derivative, DiBenzRif, have recently been described to limit P-gp ATPase activity by enhancing membrane fluidity at sub-toxic concentrations and consequently inhibiting the pump [26]. Our previous studies have demonstrated that the small molecular compound NSC23925 could reverse P-gp-mediated MDR in ovarian cancer by stimulating P-gp ATPase activity [27]. Furthermore, we evaluated the effects of NSC23925 on preventing the development of MDR in osteosarcoma and in ovarian cancer. Our studies noted that NSC23925 may prevent the development of MDR by specifically inhibiting the overexpression P-gp in both osteosarcoma and ovarian cancer [7, 28]. In addition, we confirmed that the small molecule inhibitors A-770041 and NSC77037 may function to reverse P-gp-mediated chemotherapy drug resistance [23, 29]. Though these inhibitors have been reported to reverse MDR *in vitro* and *in vivo* when combined with anticancer drugs, they have yet to be proven useful in the clinical setting.

Recently, the CRISPR-Cas9 system, a novel genome editing tool, has been implemented in a multitude of model organisms and cell types [30]. The CRISPR-Cas9 system uses Cas9, which complexes with single guided RNA (sgRNA), to cleave DNA 3-4 base pairs upstream of a protospacer-adjacent motif (PAM) and generate double-strand breaks (DSBs) in a sequence-specific manner [30]. The DSBs are then repaired either by non-homologous end joining (NHEJ)-mediated error-prone DNA repair or homologous directed repair (HDR)-mediated error-free DNA repair [30]. The former repair can generate small insertion and deletion mutations at the target sites. These mutations can disrupt and abolish the function of target genes or genomic elements. HDR-mediated error-free DNA repair requires a homology-containing donor DNA sequence as the repair template, which leads to precise gene correction or replacement [30]. It is evident that the CRISPR-Cas9 genome editing technology has revolutionized the field of genetic engineering and holds the potential to overcome many of the limitations of earlier techniques to carry out deletions, insertions, translocations, and inversions at specific sites in the DNA of cells [30].

Due to the controversial discussions on the relevance of P-gp expression in osteosarcoma, in this study, we first explore the correlation between P-gp expression and osteosarcoma prognosis through a meta-analysis of published case-control studies. Then, we adopted the CRISPR-Cas9 system to specifically inhibit *ABCB1* at the DNA level in osteosarcoma MDR cells, and further determined the effects of *ABCB1* knockout on reversing drug resistance in osteosarcoma MDR cells.

## RESULTS

### Results of meta-analysis

#### Eligible studies

**Figure 1:**
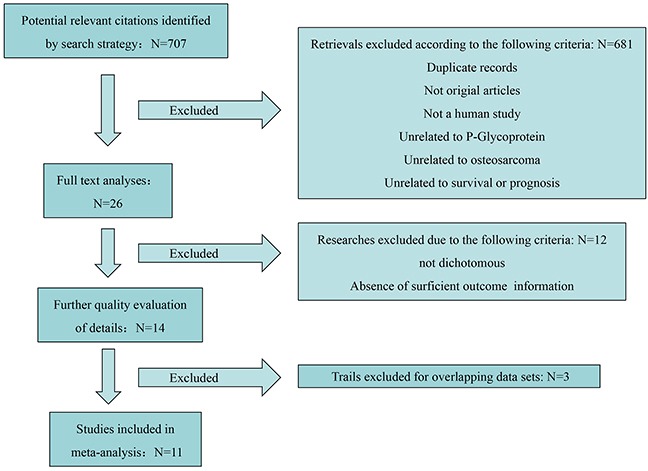
Flow diagram of the study selection process

A total of 707 studies were identified after searching in PubMed, Embase and Web of Science for publications on prognostic role of P-gp expression in osteosarcoma. The titles, publication types and abstracts were initially evaluated and the full texts were further reviewed. Finally, 11 studies that met the inclusion criteria were considered qualified for the present meta-analysis. Figure 1 showed the flow diagram of candidate study selection in our study.

#### Characteristics of included studies

We collected the essential Data from the enrolled 11 studies which were conducted between 1995 and 2016. A total of 723 participants from different territories involving the United States, Dermark, Italy, Korea, Japan and Canada. The sample size of the included study ranged from 19 to 149 patients. Immunohistochemistry (IHC) was widely applied to detect the expression of P-gp. Included studies in this meta-analysis referred to evaluate P-gp expression for prognostic outcome in osteosarcoma. The main features of these 11 studies were summarized in Table 1.

**Table 1:** Characteristics of the studies included in this meta-analysis

Author	Publication year	Origin of population	cases	Test method	Follow-up (years)
Gao Yan [11]	2016	United Sates	57	IHC	5
Sorensen [12]	2008	Denmark	116	IHC	5
Scotlandi [13]	2005	Italy	80	IHC	5
Ferrari [14]	2004	Italy	19	IHC	3
Serra [15]	2003	Italy	149	IHC	5
Park [16]	2001	Korea	35	IHC	5
Hornicek [17]	2000	United Sates	33	IHC	5
Yammamoto [18]	2000	Japan	28	IHC	5
Gorlick [19]	1999	United Sates	53	IHC	10
Chan [20]	1997	Canada	61	IHC	5
Baldini [21]	1995	Italy	92	IHC	5

IHC: Immunohistochemistry.

#### Survival associated with P-gp expression in osteosarcoma

**Figure 2:**
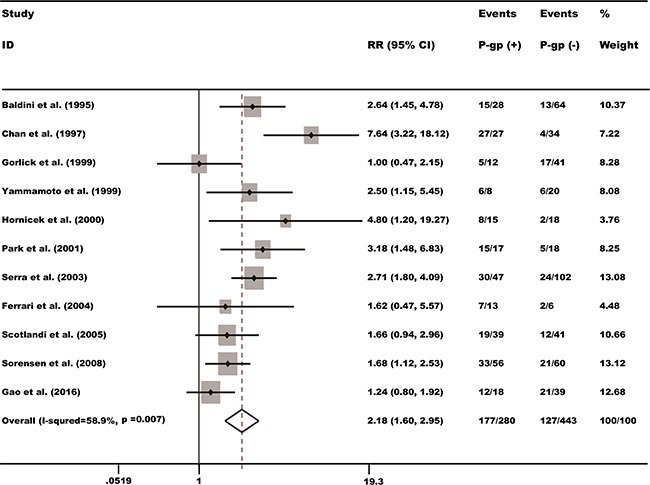
P-gp expression and overall survival rate of osteosarcoma patients

For studies evaluating survival outcome, a random effect model was applied to calculate the pooled risk ratios (RR) and its 95% confidence intervals (95% CIs) because of the significant heterogeneity had been found in the 11 cohorts (I^2^ = 58.9%, *P* = 0.007). The result showed that high level of P-gp may predict poorer survival, with the pooled RR being 2.18 (95% CI: 1.61-2.95, *P* = 0.000) (Figure 2).

#### Publication bias

Potential publication bias was assessed by Begg's funnel plot and Egger's test [31, 32]. Among 11 cohorts evaluating survival outcome, no obvious asymmetry was observed in Begg's funnel plots, and the Begg's test results also showed no potential publication bias (*P* = 0.213 > 0.05). The Egger's test results also showed no potential publication bias (t =1.14, *P* =0.282>0.05).

#### Sensitivity analysis

Sensitivity analysis investigates the influence of each individual study on the overall meta-analysis estimate, which computes the pooled HRs by omitting one study in each turn. The results of sensitivity analysis show whether the studies are convincing and stable. In the leave-one-out sensitivity analyses for P-gp expression in osteosarcoma, it demonstrated that all data assessing the prognostic role of high P-gp expression in patients with osteosarcoma were stable as the endpoint.

### Results of in vitro studies

#### Transfection of *ABCB1* sgRNA-Cas9-GFP significantly inhibits P-gp expression

**Figure 3:**
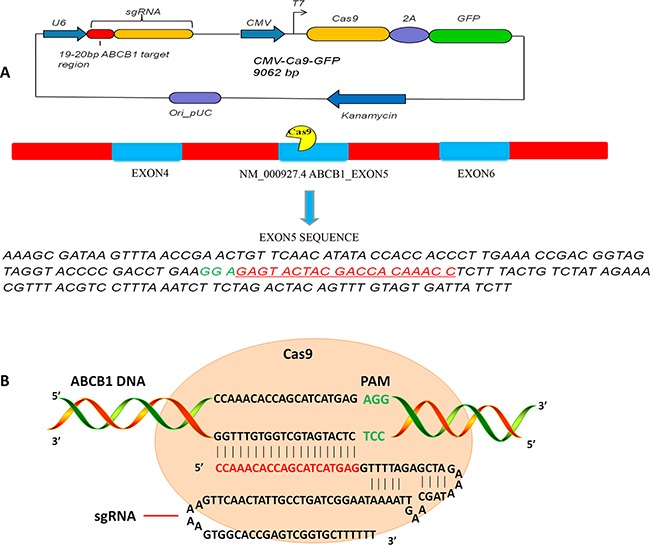
**A.** Schematic of U6 ABCB1 sgRNA-CMV Cas9-GFP expression cassette in the single plasmid system. GFP is co-expressed from the same mRNA as the Cas9 protein via a 2A peptide linkage, which enables tracking of transfection efficiency. The exon of *ABCB1* selected for guide RNA design is located on the fifth coding exon. The human U6 promoter is used to drive sgRNA expression, while the CMV promoter drives expression of Cas9 and GFP proteins. The position of the frame shift that CRISPR-Cas9 knocks out is located at the fifth exon within the *ABCB1* gene (NM_000927.4 *ABCB1*). The red font is the 20 bp for sgRNA, and AGG (green font) is the sequence of PAM. **B.** Schematic structure of CRISPR-Cas9 system functions on targeting the *ABCB1* gene. The core components of the CRISPR-Cas9 system are a nuclease Cas9 and a single guided RNA (sgRNA). The CRISPR-Cas9 system uses Cas9, which complexes with sgRNA to cleave target DNA and generate double-strand breaks (DSBs) in a sequence-specific manner about 3-4 base pairs upstream of a protospacer adjacent motif (PAM). The red font is the 20 bp guide sequence for sgRNA, and AGG (green font) is the sequence of PAM. The black font in sgRNA sequence is the commonly used sequence for tracrRNA.

**Figure 4:**
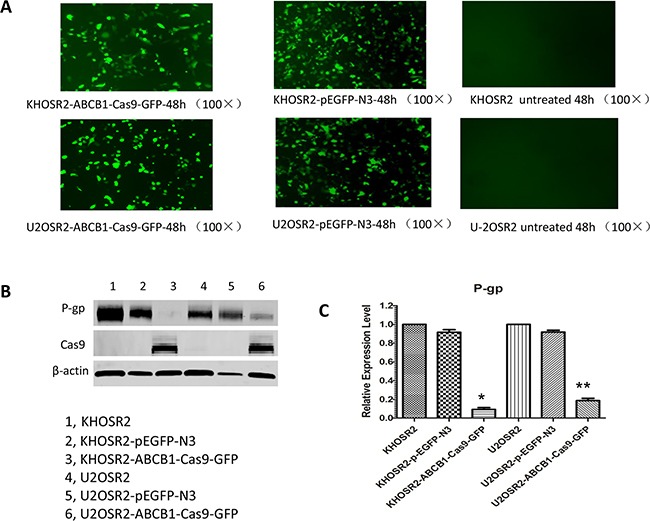
**A.** Fluorescence analysis showed that GFP was detected in KHOSR2- pEGFP-N3, KHOSR2-*ABCB1*-Cas9-GFP, U-2OSR2-*ABCB1*-Cas9-GFP, and U-2OSR2-pEGFP-N3 cells, which suggests that KHOSR2 and U-2OSR2 cells were successfully transfected with *ABCB1*-Cas9-GFP or pEGFP. **B.** Western blotting analysis confirmed that P-gp protein expression was significantly inhibited in KHOSR2 and U-2OSR2 cells transfected with *ABCB1*-Cas9-GFP. Furthermore, Cas9 protein was expressed in KHOSR2 and U-2OSR2 cells transfected with *ABCB1*-Cas9-GFP. **C.** P-gp expression was repressed 11.34 ± 1.93 fold (*p < 0.01) and 5.80 ± 1.25 fold (**p < 0.05) in KHOSR2 and U-2OSR2 cells transfected with *ABCB1*-Cas9-GFP, respectively.

The CRISPR-Cas9 and green fluorescent protein (GFP) fusion protein expression vector U6gRNA-Cas9+2A-GFP guide by *ABCB1* sgRNA was abbreviated as *ABCB1-*Cas9-GFP (Figure 3). To determine the transfection efficiency of *ABCB1*-Cas9-GFP or pEGFP-N3 plasmids into KHOSR2 and U-2OSR2 cells, fluorescence expression was evaluated by a fluorescence microscope. As illustrated in Figure 4A, GFP was detected in KHOSR2-pEGFP-N3, KHOSR2-*ABCB1*-Cas9-GFP, U-2OSR2-pEGFP-N3, and U-2OSR2-*ABCB1*-Cas9-GFP cells, which suggested that KHOSR2 and U-2OSR2 cells were successfully transfected with *ABCB1*-Cas9-GFP or pEGFP-N3.

CRISPR-Cas9 precisely enables specific genomic locus manipulation by providing sgRNA. To evaluate whether CRISPR-Cas9 complexed with *ABCB1* sgRNA could inhibit P-gp expression, Western blotting was performed. The results demonstrated that P-gp protein expression was significantly inhibited in KHOSR2 cells transfected with *ABCB1*-Cas9-GFP. P-gp expression of KHOSR2 was repressed 11.34 ± 1.93 fold (*p* < 0.01) (Figure 4B & 4C). In the U-2OSR2 cells transfected with *ABCB1*-Cas9-GFP, P-gp expression was also significantly inhibited. P-gp expression of U-2OSR2 was repressed 5.80 ± 1.25 fold (*p* < 0.05) (Figure 4B & 4C). Furthermore, as expected, Cas9 protein was expressed in KHOSR2 and U-2OSR2 cells transfected with *ABCB1*-Cas9-GFP (Figure 4B). These data revealed that P-gp expression was efficiently repressed in MDR osteosarcoma cell lines transfected with *ABCB1*-Cas9-GFP.

#### Knockout of *ABCB1* by CRISPR-Cas9 restores MDR cell sensitivity to doxorubicin

**Figure 5:**
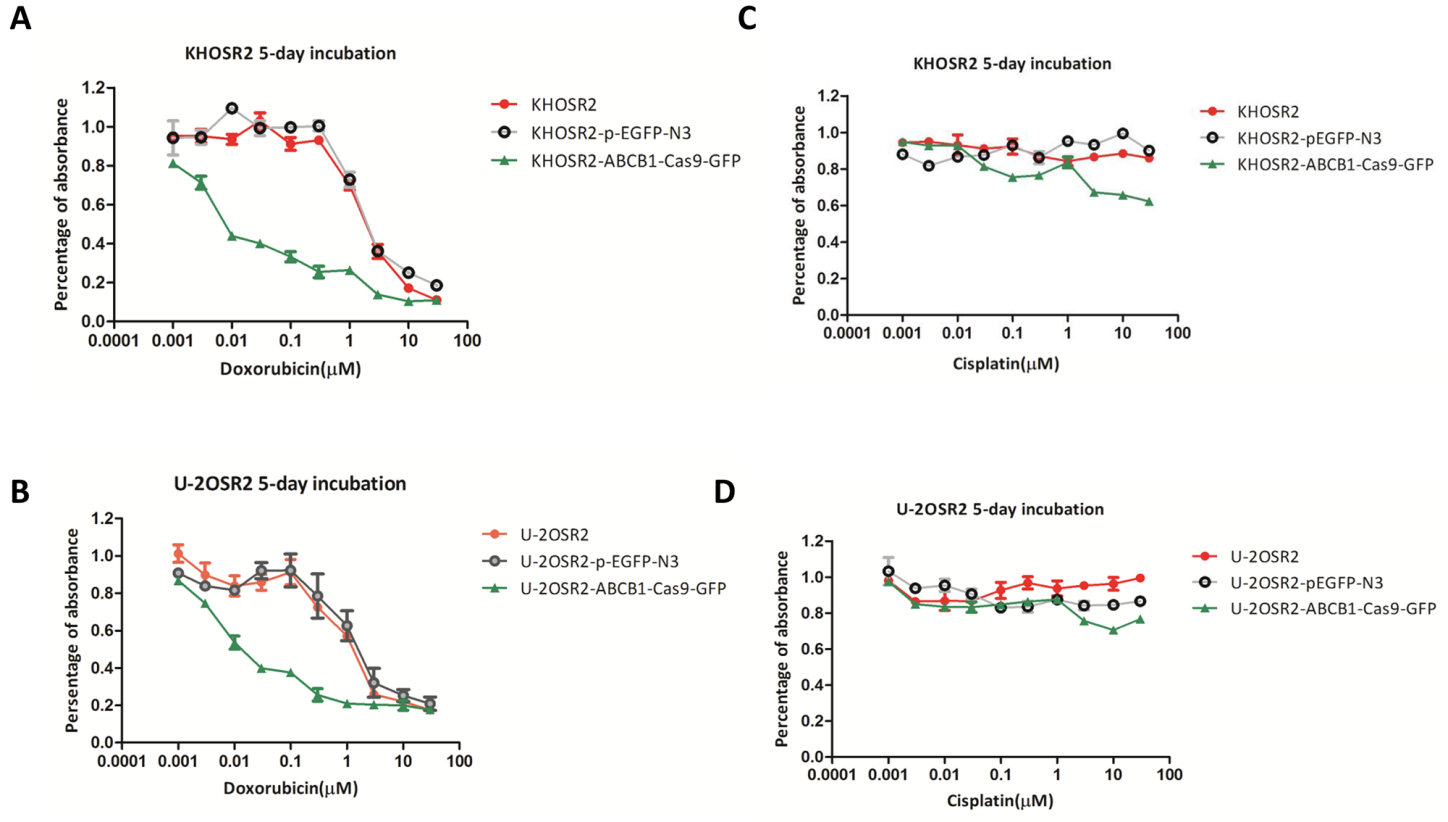
After *ABCB1* was knocked out by CRISPR-Cas9, doxorubicin exhibited an increase in anti-proliferative activity in MDR osteosarcoma cell lines in a dose-dependent manner **A, B.** while cisplatin showed no significant difference in anti-proliferative activity in the *ABCB1* knockout cells compared with that of the control cells **C, D.** In the KHOSR2 cells alone, KHOSR2 cells transfected with pEGFP-N3, and KHOSR2-*ABCB1*-Cas9-GFP cells, the IC50 of doxorubicin was 1.71 μM, 1.43 μM, and 0.05 μM, respectively. Likewise, the U-2OSR2 cells alone, U-2OSR2 transfected with pEGFP-N3, and U-2OSR2-*ABCB1*-Cas9-GFP cells, the IC50 of doxorubicin was 0.95 μM, 1.25 μM, and 0.02 μM respectively.

After *ABCB1* was knocked out by CRISPR-Cas9, we found that doxorubicin exhibited an increase in anti-proliferative activity in KHOSR2-*ABCB1*-Cas9-GFP and U-2OSR2-*ABCB1*-Cas9-GFP cells in a dose-dependent manner, while cisplatin showed no significant difference in anti-proliferative activity in the *ABCB1* knockout cells compared with that of the control cells (Figure 5). Notably, when the delivery of doxorubicin concentration was 1.0 μM, growth inhibition was observed in KHOSR2 cells with or without transfection with pEGFP-N3, and when the delivery of doxorubicin concentration was 0.3 μM, growth inhibition was detected in U-2OSR2 cells with or without transfection with pEGFP-N3. In the untransfected KHOSR2 cells and the KHOSR2 cells transfected with pEGFP-N3, the IC50 of doxorubicin was 1.71 μM and 1.43 μM, respectively, which was reduced to 0.05 μM when the cells were transfected with *ABCB1*-Cas9-GFP. Likewise, the MDR cell line U-2OSR2 and U-2OSR2 cells transfected with pEGFP-N3 displayed a similar trend-the IC50 of doxorubicin was 0.95 μM and 1.25 μM, respectively, which decreased to 0.02 μM when the cells were transfected with *ABCB1*-Cas9-GFP.

## DISCUSSION

In the present meta-analysis, we show that high expression of P-gp could predict poor survival in patients with osteosarcoma. We further demonstrated that expression of P-gp can be efficiently blocked by the CRISPR-Cas9 system and inhibition of *ABCB1* was associated with reversing drug resistance in osteosarcoma MDR cell lines (KHOSR2 and U-2OSR2) to doxorubicin. However, down regulation of P-gp had no effect on chemosensitivity to cisplatin in the *ABCB1* knockout cells compared with the control cells.

Previously, we observed that MDR1 siRNA loaded dextran nanoparticles can efficiently suppressed P-gp expression in drug resistant osteosarcoma cell lines. However, RNAi based techniques can achieve only temporary and partial knockdown of transcribed mRNA, but not genomic DNA [9]. In this study, we adopted the CRISPR-Cas9 system to knockout *ABCB1* in osteosarcoma MDR cell lines. The position of *ABCB1* sgRNA target is located on the fifth exon of the *ABCB1* gene. CRISPR-Cas9 mediates cleavage of targets on DNA sites that are complementary to the 5′-20 nt region of the sgRNA that lies next to a PAM sequence. Compared to RNAi, the advantages of the CRISPR-Cas9 system include the fact that CRISPR-Cas9 is an exogenous system that does not compete with endogenous processes and that it functions at the DNA level to target transcripts, which results in knockdown or complete elimination of gene function [30]. Furthermore, the mechanism of CRISPR-Cas9 that directly blocks transcription is distinct from that of RNAi, for which knockdown of gene expression requires the destruction of already transcribed mRNAs prior to their translation [33, 34]. In addition, CRISPR-Cas9 could provide a larger targetable sequence space in which promoters of the gene may also be targeted [30]. Thus, CRISPR-Cas9 is a novel genome editing tool for switching gene expression at the DNA level [30].

CRISPR-Cas9 guided gene targeting is highly specific [33, 35]. To evaluate the specificity of CRISPR-Cas9 on a genome-wide scale, scientists performed whole-transcriptome shotgun sequencing (RNA-seq) of Cas9-transformed cells with and without sgRNA co-expression [33]. In the presence of the sgRNA targeted to red fluorescent protein (mRFP), the mRFP transcript was the sole gene that exhibited a decrease in abundance [33]. Furthermore, scientists also performed RNA-seq on cells with different sgRNAs that target different genes. None of these experiments showed significant changes in other genes besides the target gene [33]. In our study, the results showed that CRISPR-Cas9 guided by *ABCB1* sgRNA markedly decreased P-gp expression in osteosarcoma MDR cell lines, whereas pEGFP-N3 had no effect on KHOSR2 or U-2OSR2 cell lines. Knockout of *ABCB1* by CRISPR-Cas9 restored the sensitivity of osteosarcoma MDR cell lines to doxorubicin, but not to cisplatin. These studies imply that sgRNA guided gene targeting and regulation is highly specific and *ABCB1* sgRNA guided CRISPR-Cas9 can specifically knock out *ABCB1*.

Robust gene editing has been observed in both reporter genes and endogenous genes by CRISPR-Cas9 system [36]. CRISPR-Cas9 is capable of inducing loss of function (LOF) and gain of function (GOF) mutations *in vitro* and *in vivo*. In our study, by delivering the combination of *ABCB1* sgRNA and Cas9 with Lipofectamine® 3000 Reagent, P-gp protein expression was significantly silenced. There are several factors that affect repression efficiency. Firstly, the DNA carrier could influence the transfection efficiency and gene expression. In some studies, common lentiviral constructs were used to express both Cas9 and sgRNAs to achieve stable long term gene knockdown [36]. They observed 5- to 15-fold repression of both reporter genes and endogenous genes in human [36]. In our study, transfection of sgRNA-Cas9 into KHOSR2 and U-2OSR2 cells was performed with Lipofectamine® 3000 Reagent, and P-gp expression was repressed 5.80- to 11.34-fold. This is comparable to the efficiency of existing gene editing techniques, such as RNAi or transcription activator-like effector nucleases (TALENs) [30, 36]. Secondly, it is important for silencing efficiency that the location of the sgRNA target sequence be adjacent to the gene [33]. In a study, scientists noted that repression by CRISPR-Cas9 was inversely correlated with the target distance from the transcription start site [30]. The same results were shown by another group, and in their studies, efficient activation of endogenous genes could be achieved by three to five sgRNAs binding within a 300 bp region upstream of the transcription start site [35]. Using additional sgRNAs to target further upstream or downstream regions did not significantly improve the level of induction. Their data suggest that only a small number of sgRNAs targeting the proximal promoter is sufficient to activate endogenous genes [35]. In our study, we adopted sgRNA binding at the fifth exon of the *ABCB1* gene, which was able to knockout *ABCB1* efficiently. Interestingly, while this manuscript was in the preparation, another group has used CRISPR-Cas9 to targeting *ABCB1* in canine kidney II cell line. This study also showed canine *ABCB1* can be efficiently knocked out by CRISPR-Cas9 [37].

In summary, we demonstrated that overexpression of P-gp could predict poor survival in patients with osteosarcoma. Our finding indicates that CRISPR-Cas9 is a powerful gene editing technology that can knockout *ABCB1* successfully. *ABCB1* knockout could restore the sensitivity of osteosarcoma MDR cell lines to doxorubicin. These results suggest that the CRISPR-Cas9 system will be useful in extending the long-term efficacy of chemotherapy by reversing P-gp-mediated MDR in the clinical setting.

## MATERIALS AND METHODS

### Meta analysis

This meta-analysis was conducted in accordance with the standard guidelines of Preferred Reporting Items for Systematic Reviews and Meta-Analyses (PRISMA) 2009 Checklist (http://www.prismastatement.org/statement.htm) and Meta-analysis of Observational Studies in Epidemiology group (MOOSE) [38].

### Identification of relevant studies

Searching for relevant literatures was conducted up to March 20, 2016. Electronic sources included Pubmed (http://www.ncbi.nlm.nih.gov/pubmed), MEDLINE (http://medline.cos.com/) EMBASE (http://www.embase.com/home) and Web of Science (http://wokinfo.com/) databases. The search strategy included the following sets of key words and their combination search terms: “P-glycoprotein OR *MDR1* OR *ABCB1*”, “osteosarcoma OR malignant bone tumor OR malignant bone cancer”, and “survival OR prognosis OR outcome OR death”. We also performed a search for references of retrieved articles in order to identify other potentially eligible studies. The language was limited to English. Overlapping data from the same authors were excluded from our meta-analysis.

Two independent reviewers firstly searched potentially relevant studies by reading the titles and abstracts and then further checked by reading the full texts and assessed for inclusion. Other two senior reviewers double checked these extracted articles for a second time. Disagreements were resolved by discussion among these reviewers and consultation with another senior reviewer.

### Eligibility criteria

Studies were considered eligible according to following criteria: (i) patients with osteosarcoma was studied; (ii) the associations between P-gp expression and survival outcome of patients were investigated; and (iii) sufficient data was provided to estimate RRs and corresponding 95%CIs.

Articles were excluded if they met the following criteria: (i) reviews, case reports, comments, conference abstracts, animal studies and laboratory studies; (ii) studies of non-dichotomous data; (iii) lack of crucial information to estimate RR and 95% CI.

### Quality assessment

Two investigators critically assessed the quality of all the included studies based on the critical guidelines of the Dutch Cochrane Centre proposed by MOOSE for prognostic meta-analysis [38]. The key points of the review checklist included the following: (i) clear description of study population and origin of country, (ii) clear definition of diagnosis of osteosarcoma, (iii) clear explanation of study design, (iv) clear description of outcome assessment, (v) clear report of P-gp expression measure method, (vi) clear definition of cut-off of P-gp expression, and (vii) sufficient follow-up period. We excluded the studies without specifying any aspect concerning above so as not to compromise the quality of the meta-analysis.

### Data extraction, conversion and analysis

General characteristics of the eligible articles in the meta-analysis were collected: name of the first author, year of publication, case number, origin of population, detection methods and prognosis outcome.

RRs with their 95% CIs were extracted according to the following methods [39]. Reported results for survival in included studies were considered eligible. In most instances, the total number of observed death cases and the number of samples in each group or the valuable data provided by the authors were extracted to calculate RRs. If only Kaplan–Meier curves are available, data were extracted from the graphical survival plots to estimate the RRs following the previously described method [39]. If needed, we sought original data directly from the authors of the relevant studies. All the results extracted according to the above methods were compared, and disagreements were discussed among all the authors to resolve with consensus. The pooled RRs with their 95% CIs and *P* values were reported as the results, with an RR >1 being associated with elevated risk of mortality.

The association of P-gp expression with prognostic outcome in osteosarcoma was estimated by using RR and their associated 95% CI for each study. Heterogeneity of combined RRs was assessed by Cochran's Q test and Higgin's I^2^ statistic [40]. Heterogeneity was considered statistically significant as *P*<0.05 or I^2^ > 50%. A fixed effect model (Mantel-Haenszel test) was applied in the absence of between-study heterogeneity (*P* ≥ 0.05 or I^2^ ≤ 50 %) [41], while the random effect model (Der Simonian and Laird method) was applied if significant heterogeneity was observed (*P* < 0.05 or I^2^ > 50 %) [42].

The Begg's funnel plot and Egger's bias indicator test were used to evaluate the potential publication bias among the included studies [38,39]. *P*<0.05 in all the two-sided statistical tests was regarded as significant. No corrections were made for multiple comparisons. All analyses were conducted using the STATA package version 12.0 (Stata Corporation, College Station, Texas, USA).

### In vitro studies

#### Human osteosarcoma MDR cell lines

The osteosarcoma MDR cell lines U-2OSR2 (established by selection with doxorubicin) and KHOSR2 (established by selection with doxorubicin) were previous reported by our laboratory [9, 29, 43, 44]. These cell lines with high level of P-gp were cultured in RPMI 1640 (Life Technologies, Grand Island, NY, USA) supplemented with 10% FBS, 100 units/mL penicillin, and 100μg/mL streptomycin (Life Technologies, Grand Island, NY, USA). Cells were incubated at 37°C in 5% CO_2_-95% air atmosphere and passaged when near-confluent monolayers were achieved using trypsin-EDTA solution.

#### Drugs

Doxorubicin and cisplatin were provided by the pharmacy at the Massachusetts General Hospital Cancer Center. The stock solutions of doxorubicin were prepared according to the manufacturer's specifications and stored at −20°C.

#### CRISPR-Cas9 plasmid design and purification

The CRISPR-Cas9 and green fluorescent protein (GFP) fusion protein expression vector U6gRNA-Cas9+2A-GFP guide by *ABCB1* sgRNA (abbreviated as *ABCB1-*Cas9-GFP) was purchased from Horizon Discovery (DNA 2.0 Inc., CA, USA). GFP was co-expressed from the same mRNA as the Cas9 protein via a 2A peptide linkage, which enabled tracking of transfection efficiency. The exon of *ABCB1* selected for sgRNA design is located on the fifth coding exon (Figure 3A). The *ABCB1* sgRNA sequence is as follows: 5′-CCAAACACCAGCATCATGAG-3′ (Figure 3B). The pEGFP-N3 plasmid was purchased from Clontech Laboratories, Inc. (Mountain View, CA, USA). Plasmids were purified using QIAGEN Plasmid Mega Kits (Hilden, Germany) according to the Plasmid Purification Handbook. To determine the yield of each plasmid, DNA concentrations were determined by both UV spectrophotometry at 260 nm and quantitative analysis on an agarose gel.

#### Work flow of Lipofectamine-mediated transfection of *ABCB1* sgRNA-Cas9-GFP

Transfection of *ABCB1* sgRNA-Cas9-GFP into KHOSR2 and U-2OSR2 cells was performed with Lipofectamine® 3000 Reagent (Life Technologies, Grand Island, NY, USA) according to the manufacturer's instructions. Briefly, U-2OSR2 and KHOSR2 cells were seeded in 12-well plates at a density of 1.0×10^5^ cells/mL and 7×10^4^ cells/mL, respectively, with 1 mL of cells per well. After 24 h, Opti-MEM® Medium was used to rinse the cells three times and 1 mL of serum-free medium was added for cell culturing. Then, 1.5 μl of Lipofectamine® 3000 Reagent was diluted in 50 μl of Opti-MEM® Medium, and a master mix of 1 μg DNA in 50 μl of Opti-MEM® Medium was prepared with 2.0 μl P3000™ Reagent. Next, the diluted DNA was added to the tube of diluted Lipofectamine® 3000 Reagent (1:1 ratio). After incubation for 5 min at room temperature, the DNA-lipid complex was added to the cells. After incubation for 48 h, the positive cells successfully transfected with *ABCB1* sgRNA-Cas9-GFP plasmid were sorted by flow cytometry and the cultures were expanded for further study; untransfected cells were used as controls.

#### Fluorescence microscope observation

To observe the transfection efficiency of *ABCB1*-Cas9-GFP or pEGFP-N3 plasmid expression into the U-2OSR2 and KHOSR2 cells, fluorescence analysis used to determine the GFP expression levels in the transfected cells. Briefly, U-2OSR2 and KHOSR2 cells were seeded in 12-well plates at a density of 1.0×10^5^ cells/ml and 7×10^4^ cells/ml, respectively, with 1 mL of cells per well. The cells were then transfected with *ABCB1*-Cas9-GFP or pEGFP-N3 plasmid. After incubation for 48 h, the cells were evaluated under fluorescence. Osteosarcoma MDR cells were then visualized on a Nikon Eclipse Ti-U fluorescence microscope (Nikon Instruments, Inc., NY) equipped with a SPOTRT digital camera from Diagnostic Instruments, Inc. (Sterling Heights, MI).

#### Western blotting

After transfection of the *ABCB1*-Cas9-GFP plasmid, expression of the P-gp protein in KHOSR2 and U-2OSR2 cells was evaluated by Western blotting. Protein lysates from osteosarcoma cells were extracted using 1× RIPA Lysis Buffer (Upstate Biotechnology, Charlottesville, VA, USA). The protein concentrations were determined by Protein Assay Reagents (Bio-Rad, Hercules, CA, USA) and a SPECTRAmax Microplate Spectrophotometer from Molecular Devices (Sunnyvale, CA, USA). The primary monoclonal antibodies for ABCB1 (1:1000 dilution) and Cas9 (1:1000 dilution) were purchased from Cell Signaling Technology (Danvers, MA, USA). Secondary antibodies IRDye^®^800CW or IRDye^®^680LT were purchased from LI-COR Biosciences (Lincoln, NE, USA). Western blotting analyses were carried out as previously described^12^. Membrane signals were scanned using the Odyssey infrared imaging system and analyzed using Odyssey 3.0 software (LI-COR Biosciences, NE, USA). Relative expression values were normalized assigning the value of the cells in control groups to 1.0.

#### MTT assay

The MTT assay was performed to estimate the drug resistance profile of the tested cells to doxorubicin and cisplatin. Briefly, KHOSR2 or U-2OSR2 cells transfected with or without pEGFP-N3 or *ABCB1*-Cas9-GFP were seeded into 96-well culture plates at a density of 3×10^3^ cells per well. The cells were then treated with increasing concentrations of doxorubicin or cisplatin for five days. Afterwards, 20 μL of MTT (5 mg/mL in PBS, purchased from Sigma-Aldrich, MO, USA) was added to each well and the plates were incubated for an additional four hours. Finally, the resulting formazan product was dissolved with acid (HCl)-isopropanol and the absorbance at a wavelength of 490nm was read on a SPECTRAmax Microplate Spectrophotometer from Molecular Devices (Sunnyvale, CA, USA). Experiments were done in triplicate. Dose-response curves were fitted using GraphPad PRISM 5 software (GraphPad Software, La Jolla, CA).

#### Statistical analysis

GraphPad PRISM 5 software (GraphPad Software, La Jolla, CA) was used to statistically analyze the data. The differences between groups were also evaluated using the two-sided Student's *t*-test. Errors were SD of averaged results, *p* values <0.05 were considered statistically significant between means, and *p* values <0.01 were accepted as a significant difference between means.
